# A New V361A Mutation in *Amaranthus palmeri PPX2* Associated with PPO-Inhibiting Herbicide Resistance

**DOI:** 10.3390/plants12091886

**Published:** 2023-05-05

**Authors:** Haozhen Nie, Nick T. Harre, Bryan G. Young

**Affiliations:** 1 Shanghai Key Laboratory of Plant Functional Genomics and Resources, Shanghai Chenshan Botanical Garden, Shanghai 201602, China; 2 Department of Botany and Plant Pathology, Purdue University, West Lafayette, IN 47907, USA

**Keywords:** herbicide resistant screen, PPO-inhibiting herbicide resistance, protoporphyrinogen oxidase, resistance mechanism, target site resistance, *PPX2*, R128, ∆G210

## Abstract

Weeds resistant to PPO-inhibiting herbicides threaten the profitability of crop producers relying on this chemistry. In *Amaranthus palmeri*, mutations at G210 (∆G210) and R128 (R128G/M) of the *PPX2* gene were reported to confer PPO-inhibitor resistance. Here, *A. palmeri* samples from nine states in America, having survived a field application of a PPO-inhibitor, were genotyped to determine the prevalence of these mutations. Less than 5% of the 1828 *A. palmeri* plants screened contained the ∆G210 mutation. Of the plants lacking ∆G210, a R128 substitution was only found in a single plant. An *A. palmeri* population from Alabama without mutations at G210 or R128 had a resistance ratio of 3.1 to 3.5 for fomesafen. Of the candidate *PPX2* mutations identified in this population, only V361A conferred resistance to lactofen and fomesafen in a transformed bacterial strain. This is the first report of the V361A substitution of PPX2 conferred PPO-inhibiting herbicide resistance in any plant species. Future molecular screens of PPO-inhibitor resistance in *A. palmeri* and other species should encompass the V361A mutation of *PPX2* to avoid false-negative results.

## 1. Introduction

Protoporphyrinogen IX oxidase (PPO) is the last enzyme of heme and chlorophyll biosynthesis which converts protoporphyrinogen IX to protoporphyrin IX [1]. Inhibition of the PPO enzyme by PPO-inhibiting herbicides causes an accumulation of protoporphyrinogen IX [2] which generates high levels of singlet oxygen in the presence of light, resulting in a phytotoxic effect in sensitive plant tissue [3]. Two different nuclear genes in plants, *PPX1* and *PPX2*, encode plastid and mitochondrial PPO isozymes [4,5]. To date, mutations in the *PPX2* gene that were documented to confer resistance in weeds include a codon deletion (∆G210) in *Amaranthus tuberculatus* (waterhemp) [6] and *Amaranthus palmeri* (Palmer amaranth) [7], and substitutions at the R128 residue in *Ambrosia artemisiifolia* (R128L) [8], *A. palmeri* (R128G/M) [9], and *A. tuberculatus* (R128G/I) [10,11], and the substitution G399A in *A. palmeri* [12,13].

Dayan et al. suggested that *PPX* target-site mutations provide the best evolutionary route for development of resistance to PPO-inhibiting herbicides [14]. In crystal structure analysis of the *Nicotiana tabacum* PPO enzyme, the R98 site (named as R128 in weed species) along with L356, L372, and F392 are inhibitor binding residues, while ∆G210 destabilizes the α-8 helix causing the active site cavity lined with F467, V360, P361, L362, G422, G423, and F420 to enlarge [14,15,16]. Research investigating DNA sequences encoding PPO enzymes from multiple species reported that modifications at the 240, 245, 246, 388, 390, 451, 455, 500, or 536 position of *PPX* genes conferred resistance to PPO-inhibitors [17]. In other research, *Arabidopsis* plants expressing a double mutant PPO (Y426M + S305L) were resistant to several classes of PPO-inhibiting herbicides [18], while a V389M substitution conferred resistance to a PPO-inhibitor in a herbicide selected *Chlamydomonas reinhardtii* Dang green alga mutant (*rs-3*) [19]. Although many of the previously described mutations were generated through genetic engineering, it is conceivable that weeds could evolve resistance to PPO-inhibiting herbicides through mutations other than those documented at the R128 and G210 positions.

*A. palmeri* and *A. tuberculatus* are two problematic weed species which evolved resistance to numerous herbicide sites of action including acetolactate synthase (ALS) inhibitors, 4-hydroxyphenylpyruvate dioxygenase (HPPD) inhibitors, 5-enolpyruvylshikimate-3-phosphate synthase (EPSPS) inhibitors, photosystem II inhibitors, and PPO inhibitors [20]. *A. tuberculatus* and *A. palmeri* resistant to PPO-inhibitors (PPO-R) were first reported in Kansas in 2003 [21] and in Arkansas in 2016 [7], respectively. In previous surveys of PPO-R *A. tuberculatus* and *A. palmeri*, the ∆G210 mutation was present in nearly all resistant *A. tuberculatus* populations [22] but only 55% of resistant *A. palmeri* populations [23]. In Arkansas state survey, it was found that 106 out of 167 resistant *A. palmeri* accessions had ∆G210 or R128G/M mutations [24]. Recently, the frequency of the target-site-mutations (TSM), including the ∆G210, G399A, and R128G/M, was screened in PPO-R *A. palmeri* in mid-south US states survey. It was reported that 139 out of 147 resistant accessions had known-TSM mutations [25]. Given the notable percentage of resistant *A. palmeri* lacking known-TSM mutations, the occurrence of other point mutations in *PPX2* conferring resistance to PPO-inhibiting herbicides must be considered.

Research was conducted to find novel target-site-mutation in *PPX2* in *A. palmeri*. The frequency of ∆G210, R128G, and R128M in suspected PPO-R *A. palmeri* populations from a wide geographic region was investigated firstly. Additionally, a population from Alabama state, which is resistant to PPO-inhibiting herbicides but lacking the ∆G210 and R128G/M mutation, was selected for investigation into other PPO target-site mutations.

## 2. Results

### 2.1. ∆G210 and R128 Mutation Frequency in A. palmeri Populations

The molecular survey to determine the frequency of the ∆G210 mutation included 1828 plants from 84 locations in nine states in America (Table 1, Figure S1). The ∆G210 mutation was identified in only 4% of individuals screened with the majority of these plants (89%) being heterozygous for the trait. None of the submissions from North Carolina (NC), South Carolina (SC), or Alabama (AL) and less than 1% of plants from Mississippi (MS) carried the ∆G210 trait. Although a higher proportion of populations from TN contained individuals with ∆G210, the overall frequency of plants with ∆G210 was just 7%. In contrast, 50% of plants from AR carried the ∆G210 mutation. The results were similar to recent research in AR, which found 46 to 60% of *A. palmeri* that survived PPO-inhibiting herbicides contained the ∆G210 mutation [23]. However, the low overall frequency of ∆G210 among *A. palmeri* populations was notably different from recent research on *A. tuberculatus* accessions from five Midwestern states in which the ∆G210 mutation was identified in 125 out of 148 populations [26].

Among the 170 plants from 17 populations screened for mutations at the R128 site, only one plant had the R128G substitution. The R128M substitution was absent in all 170 individuals screened.

### 2.2. Whole Plant Dose–Response

In the whole plant dose–response assay, the PPO-R *A. palmeri* population from AL with an unknown mechanism of resistance was less sensitive to fomesafen than the PPO-S *A. palmeri* population with resistant/susceptible (R/S) values of 3.5 and 3.1 for fresh and dry weight, respectively, while PPO-R *A. palmeri* with ∆G210, R/S values were 2.4 and 2.0 (Table 2, Figure S2). Indeed, future research using controlled crosses is warranted to create a more homogeneous sample from the AL population, in order to better calculate the magnitude of resistance. Nevertheless, these results confirm that the level of resistance to fomesafen in the AL population was at least similar to that observed in the population resistant via ∆G210.

### 2.3. Investigation of Alternative Resistance Mechanisms

#### 2.3.1. Three Substitutions Were Revealed in PPO-R Resistant AL Plants

**Figure 1:**
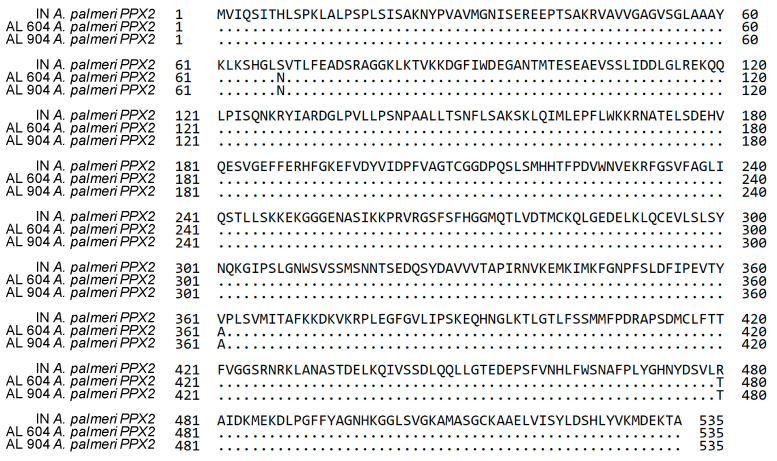
Alignment of PPX2 protein sequence of *A. palmeri* PPO-susceptible plant (from IN, Genbank number MH910646) and two *A. palmeri* PPO- resistant plants (from AL population, Genbank number_MH910647). Dots mean that sequences are identified with susceptible sequence. Three changed amino acids are shown, S68N, V361A, and R480T.

**Figure 2:**
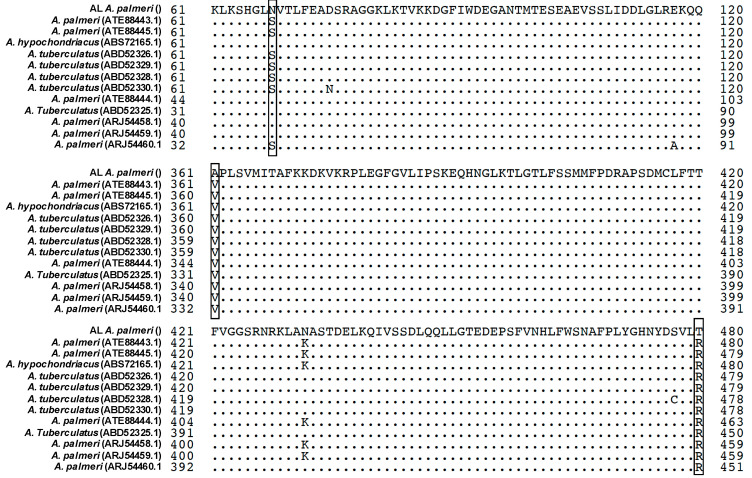
Partial alignment of PPX2 protein sequence in Alabama *A. palmeri* resistant plant and PPX2 protein sequences of Amaranthus species in NCBI data base. Query line (first line) is sequence of PPX2 protein in Alabama *A. palmeri* resistant plant. The followed twelve lines are twelve versions of PPX2 protein sequences from Amaranthus species. The candidate substitutions, S68N, V361A, and R480T, are marked in frame. Dots mean that sequences are identified with the first line.

Comparison of PPX2 sequences from the resistant AL plants with PPO-S *A. palmeri* revealed three substitutions in both AL plants: S68N, V361A and R480T (Figure 1). The amino acid conservation level associated with each of the three substitutions was determined by comparing the AL PPX2 sequence with 12 other Amaranthus plants using BLASTP on NCBI (Figure 2). The S68N substitution was present in other *A. palmeri* and A. tuberculatus sequences in the NCBI database. However, V361 and R480 are highly conserved across Amaranthus PPX2 sequences (Figure 2); thus, mutations at these residues were more likely related to PPO-inhibitor resistance in the AL population.

#### 2.3.2. V361A Substitution Was Identified to Be Associated with PPO-R Trait in Functional Complementation Analysis

**Figure 3:**
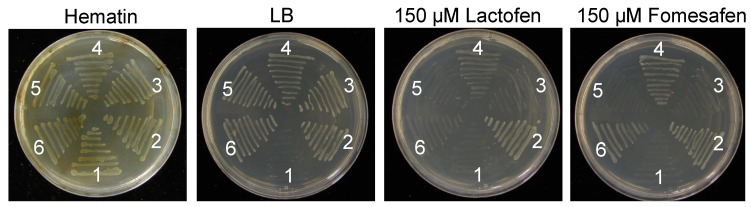
Functional complementation assay in the BT3 *HemG E. coli* strain. *E. coli* strains grew on the LB medium or LB medium with supplement of 20 µg mL^−1^ Hematin, 150 µM Lactofen, and 150 µM fomesafen, respectively. Isolates are as follows: 1, BT3 *HemG* strain untransformed control; 2, transformed with construct encoding *A. palmeri* resistant PPX2 protein with ∆G210; 3, transformed with construct encoding *A. palmeri* susceptible PPX2 protein; 4, transformed with construct encoding *A. palmeri* PPX2 protein with V361A and R480T substitutions, which is identified in Alabama palmer plant; 5, transformed with construct encoding mutagenized *A. palmeri* PPX2 protein with R480T substitution; 6, transformed with construct encoding mutagenized *A. palmeri* PPX2 protein with V361A substitution.

Growth of BT3 *E. coli* transformed with the V361A and R480T construct was observed in media supplemented with lactofen and fomesafen, indicating resistance to PPO-inhibiting herbicides (Figure 3). To further clarify the resistance conferring mutation, site-directed mutagenesis was used to create separate constructs for the V361A and R480T substitutions. Growth on lactofen and fomesafen supplemented media was observed from BT3 *E. coli* transformed with the V361A construct but not the R480T construct. Thus, this assay demonstrated that the V361A substitution in PPX2 of AL *A. palmeri* conferred resistance to PPO-inhibiting herbicides.

#### 2.3.3. The Resistant Level of PPX2 with V361A Substitution Is Less Than PPX2∆G210 in *E. coli* System

**Figure 4:**
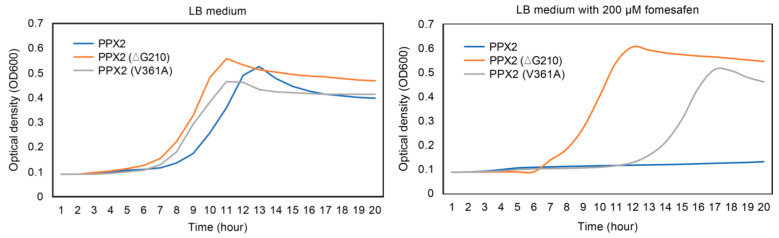
Growth curves of BT3 *hemG* mutant transformed with different *PPX2* vectors grown in LB medium or LB medium with 200 µM fomesafen. BT3 *hemG* mutant transformed susceptible *PPX2* is show in blue line; BT3 *hemG* mutant transformed *PPX2* with V361A is show in grey line; and BT3 *hemG* mutant transformed *PPX2* with ∆G210 is show in orange line.

To compare the resistance level of PPX2 with ∆G210 and PPX2 with V361A, a dose–response testing was performed in BT3 hemG mutant strain. At 200 µM fomesafen, BT3 hemG transformed PPX2 did not grow well. Interestingly, BT3 hemG transformed PPX2 with ∆G210 grew much faster than BT3 hemG transformed PPX2 with V361A, suggesting that PPX2 with V361A substitution confers a lower resistance level than PPX2_∆G210_ (Figure 4).

## 3. Discussion

### 3.1. Low Frequency of ∆G210 and R128 Mutation in PPO-R A. palmeri Survey

The discovery of PPO-R *A. palmeri* is relatively new in the herbicide-resistant weed arena [18] and based on previous molecular screens of populations from AR [23,24] and mid-south US survey [25], it might be expected that ∆G210 is the predominant underlying genetic basis for resistance. However, these results indicate the frequency of this mutation in *A. palmeri* having survived treatment with a PPO-inhibiting herbicide to be less than 5%. Moreover, the contribution of R128 substitutions to the observed field resistance of these samples was negligible. The relatively low occurrence of the R128 and ∆G210 mutations suggests that other mechanisms of resistance to PPO-inhibiting herbicides may be present among *A. palmeri* populations. On the other hand, the low frequency of these mutations in the *A. palmeri* populations may in part be caused by some sampling of PPO-S plants.

### 3.2. V361A Substitution in PPX2 Confers PPO-R Trait in PPO-R A. palmeri

In the PPO-R *A. palmeri* population from AL, V361A substitution was found in PPX2 gene. Then, the V361A substitution was identified to be associated with PPO-R trait in functional complementation analysis using *E. coli* system. According to the crystal structure of PPO, the V361 residue lies in the β-12 sheet near the inhibitor binding cavity [12]. Work elsewhere reported V360 (numbered 361 in this study), F467, P361, L362, G422, G423, and F420 constitute a hydrophobic pocket near the active site cavity and the PPO protein with ∆G210 increases the volume of this cavity by approximately 50% [10]. Consequently, the larger cavity supplies enough space for both substrate and inhibitor binding [10]. Further research is necessary to determine the structural consequences of the V361A substitution on substrate and inhibitor binding properties in order to clarify how this mutation endows resistance to PPO-inhibitors.

In functional complementation analysis in *BT3 hemG,* the resistance level of *PPX2* with V361A substitution was less than *PPX2_∆G210_* shown in the bacterial growth curve. However, in the previous whole plant dose–response assay, the AL population showed at least a similar resistance level with the population via ∆G210. We proposed another undocumented resistance mechanism must be present in this AL population. Pure genetic lines of these resistant plants must be created and further tested against PPO-inhibiting herbicides to elucidate the resistance mechanism.

This work documents a new target-site mutation in PPO-R *A. palmeri*, the frequency and distribution of V361A remains unknown. Numerous point mutations in other plant species were shown to confer resistance to PPO-inhibitors. Thus, it is plausible additional mutations either have yet to evolve in PPO-R *A. palmeri* or already exist in a population yet to be genotyped.

## 4. Materials and Methods

### 4.1. Plant Material and Initial PPO-Inhibitor Resistance Screening

Leaf samples from *A. palmeri* plants, which survived in the field after PPO-inhibiting herbicide treatment, were collected, 5 to 20 plants per location, and 84 locations across nine states in America in 2016 summer with the majority of samples collected in Mississippi and Tennessee (Table 1). DNA was extracted from leaf samples using a modified CTAB method [27]. The presence of ∆G210 was determined using a TaqMan qPCR assay [28]. modified for use in *A. palmeri* [9]. Following testing for ∆G210, plants from 17 populations lacking ∆G210 were selected for genotyping to evaluate the occurrence of the R128G or R128M mutations. Two derived cleaved amplified polymorphic sequences (dCAPS) assays were employed to determine the presence or absence of R128G and R128M, as described in previous research [9].

### 4.2. Whole Plant Dose Response to Fomesafen

Seeds from an *A. palmeri* population lacking the ∆G210 and R128 mutations were collected from a suspected PPO-R population in Alabama (AL) with a history of multiple fomesafen applications. Greenhouse dose–response experiments were designed to determine the level of fomesafen resistance in the AL population compared with PPO-inhibitor susceptible (PPO-S) and PPO-R via ∆G210 populations from Indiana. *A. palmeri* seedlings with 6 to 8 leaves were sprayed with fomesafen (Flexstar^®^, Syngenta Crop Protection LLC, Greensboro, NC, USA) at 0, 0.125, 0.396, 1.25, 3.96, 12.5, 39.6, 125, 396, and 1250 g ai ha^−1^ using a spray chamber. Crop oil concentrate (Prime Oil, Winfield Solutions, LLC, St. Paul, MN, USA) at 1.0% *v*/*v* was included with each herbicide treatment.

*A. palmeri* injury was visually evaluated and plant shoots were harvested for determination of fresh and dry weight at 14 days after herbicide application. The experiment included ten replicates and was conducted twice. As a result of a non-significant run by treatment interaction in the ANOVA, data were pooled over both runs of the experiment. Shoot fresh and dry weight calculated as a percentage of the non-treated control were analyzed using the three-parameter (Equation (1)) and four-parameter log-logistic model (Equation (2)), respectively, using R software and the DRC package [29]:

(1) $$f\left(x\right)=\frac{d}{1+\exp\;(b\left(\log\left(x\right)-\log(e)\right))}$$

(2) $$f\left(x\right)=c+\frac{d-c}{1+\exp\;(b\left(\log\left(x\right)-\log(e)\right))}$$

where *e* is the fomesafen dose necessary to reduce shoot fresh or dry weight by 50% (GR_50_), *b* is the slope of the curve around *e*, *d* is the upper limit, and *c* is the lower limit [30]. Differences in dose response curves were determined by comparing standard errors of GR_50_ estimates. The level of resistance was determined by calculating a resistant/susceptible (R/S) ratio using GR_50_ values for fresh and dry weights (GR_50_ for resistant population divided by GR_50_ for susceptible population).

### 4.3. Investigation of Alternative Resistance Mechanisms

#### 4.3.1. cDNA Clone Amplification and Protein Alignment Analysis

To further investigate the resistance mechanism in the AL population, cDNA from the *PPX2* gene was sequenced in two plants that survived at 1250 g ha^−1^ fomesafen in the dose–response experiment. RNA from PPO-S IN *A. palmeri* and PPO-R AL plants was isolated from 100 mg of leaf tissue with a TRIzol kit (Invitrogen, Carlsbad, CA, USA). MLV reverse transcription enzyme (Promega, Madison, WI, USA) was used for cDNA transcription. *PPX2* CDS was amplified using forward primer 5′-ATGGTAATTCAATCCATTACCCACC-3′ and reverse primer 5′-TTACGCGGTCTTCTCATCCATCTTCAC-3′ and cloned into a pCR-blunt vector using the Zero Blunt PCR Cloning kit (Invitrogen, Carlsbad, CA 92008, USA) for sequencing. Sequences were submitted to Genebank (IN *A. palmeri* PPX2 is MH910646 and AL *A. palmeri* PPX2 is MH910647). Sequence alignment was performed using BLASTP from NCBI.

#### 4.3.2. Functional Complementation Analysis

In order to determine if the V361A and R480T substitutions confer resistance to PPO-inhibiting herbicides, a functional complementation assay was conducted with *E. coli* (BT3 *HemG* strain) grown in the presence and absence of fomesafen and lactofen using previously described methods [6,8]. A shortened version of *PPX2 (S-PPX2L)* CDS, starting at 91 bp, was amplified and cloned into a pET28a protein expression vector. PCR primers included PPX-EcoR1-F (5′-CGGAATTCCAGGAATAAGTAATGGGCAACATTTCTGAGCGAG-3′) and PPX-Sal1-R (5′-ACGCGTCGACTTACGCGGTCTTCTCATCCATCTTCAC-3′). Three variants of *A. palmeri S-PPX2L* were cloned: PPO-S, PPO-R with the ∆G210 mutation, and AL PPO-R with V361A and R480T mutations. Using site-directed mutagenesis (Q5 Site-Directed Mutagenesis Kit, New England BioLabs, Ipswich, MA, USA), the V361A and R480T substitutions were introduced into susceptible *S-PPX2L* genes to allow for comparison of the two mutations. The five resulting *S-PPX2L* constructs were transferred into a BT3 *hemG* strain. Bacterial growth was evaluated on LB medium, LB medium with 20 µg mL^−1^ hematin (Sigma, St. Louis, MO, USA, Lot#SLBR1567V), LB medium with 150 µM lactofen (Chemodex, Worksop, UK, CDX-L0013), and LB medium with 150 µM fomesafen (Sigma, Lot#SZBE077XV). Hematin, lactofen, and fomesafen were prepared in DMSO as 1000X stock and were then spread on top of the solidified LB medium plates.

#### 4.3.3. Comparison of the Resistance Level between G210 Deletion and V361A in PPX2 in *E. coli* System

Bacterial strains with three kinds of vectors, including *PPX2*, *PPX2* with G210 deletion, and *PPX2* with V361A substitution, were streaked on the LB medium plate supplemented with 100 mg mL^−1^ kanamycin to isolate single clone, respectively. Four clones for each strain were inoculated into four individual tubes with 3 mL liquid of LB medium supplemented with 100 mg mL^−1^ kanamycin overnight. The 100 µL culture was diluted into 900 µL of LB medium. Then, 2 µL of dilute culture was added to 180 µL of LB medium supplemented with 100 mg mL^−1^ kanamycin and technical grade fomesafen at a range of concentration for final dose–response testing in the 96-well plate. The fomesafen concentration range was 0, 25, 50, 100, 200, 400, 800, and 1600 µM. All of the inoculated cultures were incubated in 37 °C. The OD_600_ absorbance was measured every 15 min for 20 h using microplate reader (Infinite 200 Pro). The growth curve was drawn with the data collected from 200 µM fomesafen.

## Figures and Tables

**Table 1:** Occurrence of ∆G210 mutation in *A. palmeri* populations collected from 9 states in 2016.

State	Locations ^a^		Individual Genotypes ^b^		
	Total	Resistant	Homozygous Resistant	Heterozygous Resistant	Susceptible
Alabama	6	0	0	0	83
Arkansas	3	3	1	6	7
Illinois	1	1	0	2	3
Indiana	2	0	0	0	6
Missouri	3	1	2	1	11
Mississippi	32	2	0	5	808
North Carolina	5	0	0	0	44
South Carolina	3	0	0	0	28
Tennessee	29	10	5	54	762

^a^ Each location represents an accession from a unique field site and locations were considered resistant if at least one individual contained the ∆G210 mutation. ^b^ Genotype totals reflect the sum across all individuals and locations within a given state. Individuals with two, one or no alleles containing the ∆G210 mutation were labeled homozygous resistant, heterozygous resistant, and susceptible, respectively.

**Table 2:** Fomesafen rates that reduced growth of susceptible and resistant *A. palmeri* by 50% (GR_50_) and resistant/susceptible (R/S) ratios.

Data	Population	GR_50_ Estimate		
		Value	Standard Error	R/S Value
Fresh weight		g ha^-1^		
	Susceptible	4.0	0.8	
	Resistant with ∆G210	8.9	2.4	2.4
	AL resistant	13.9	3.4	3.5
Dry weight	Susceptible	1.8	0.4	
	Resistant with ∆G210	3.6	1.6	2.0
	AL resistant	5.6	2.1	3.1

## Data Availability

The data presented in this study are openly available in NCBI database: IN *A. palmeri* PPX2 is MH910646 and AL *A. palmeri* PPX2 is MH910647.

## Supplementary Materials

The following supporting information can be downloaded at: https://www.mdpi.com/article/10.3390/plants12091886/s1, Figure S1: Map of nine states in USA for PPO resistant Palmer survey; Figure S2: Dose response curve of dry weight in whole plant dose response assay.
