# Supplementary material for: A New V361A Mutation in Amaranthus palmeri PPX2 Associated with PPO-Inhibiting Herbicide Resistance
Source: Plants (Basel). 2023 May 5;12(9):1886. doi: 10.3390/plants12091886 (PMC10181388; doi:10.3390/plants12091886)
Supplement: Supplementary file 1 [file plants-12-01886-s001.zip › plants-2242567-Supplementary.pdf]

Supplemental Figures

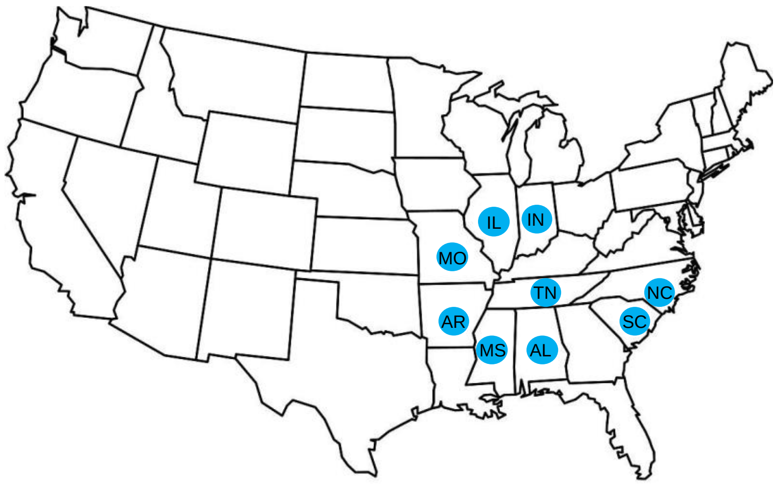

Figure S1. Map of nine states in USA for PPO resistant Palmer survey.

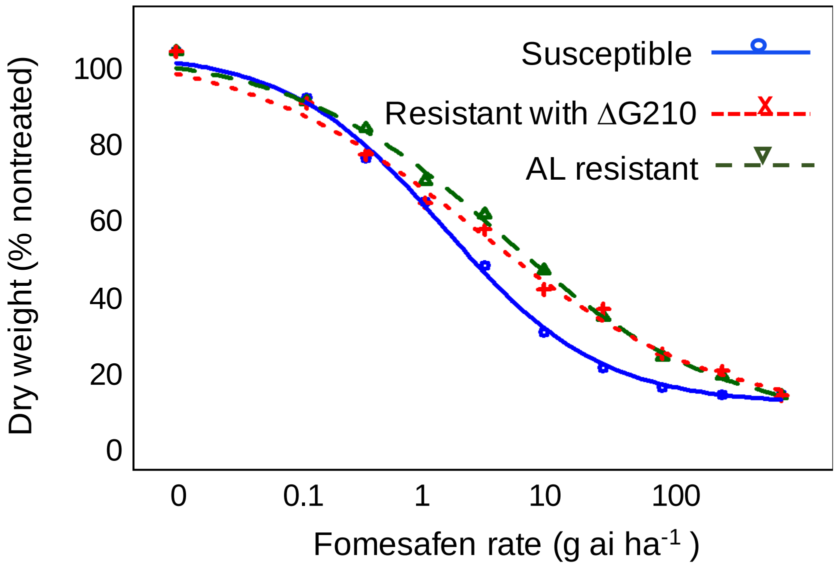

Figure S2. Dose response curve of dry weight in whole plant dose response assay.
